# Explainable artificial intelligence for spectroscopy data: a review

**DOI:** 10.1007/s00424-024-02997-y

**Published:** 2024-08-01

**Authors:** Jhonatan Contreras, Thomas Bocklitz

**Affiliations:** 1 Institute of Physical Chemistry (IPC) and Abbe Center of Photonics (ACP), Friedrich Schiller University Jena, Member of the Leibniz Centre for Photonics in Infection Research (LPI), Helmholtzweg 4, 07743 Jena, Germany; 2 Leibniz Institute of Photonic Technology, Member of Leibniz Health Technologies, Member of the Leibniz, Centre for Photonics in Infection Research (LPI), Albert Einstein Straße 9, 07745 Jena, Germany; 3 Institute of Computer Science, Faculty of Mathematics, Physics & Computer Science, University Bayreuth, Universitaetsstraße 30, 95447 Bayreuth, Germany

**Keywords:** Explainable artificial intelligence, Interpretability, Spectroscopy, Machine learning, Raman spectra, SHAP

## Abstract

Explainable artificial intelligence (XAI) has gained significant attention in various domains, including natural and medical image analysis. However, its application in spectroscopy remains relatively unexplored. This systematic review aims to fill this gap by providing a comprehensive overview of the current landscape of XAI in spectroscopy and identifying potential benefits and challenges associated with its implementation. Following the PRISMA guideline 2020, we conducted a systematic search across major journal databases, resulting in 259 initial search results. After removing duplicates and applying inclusion and exclusion criteria, 21 scientific studies were included in this review. Notably, most of the studies focused on using XAI methods for spectral data analysis, emphasizing identifying significant spectral bands rather than specific intensity peaks. Among the most utilized AI techniques were SHapley Additive exPlanations (SHAP), masking methods inspired by Local Interpretable Model-agnostic Explanations (LIME), and Class Activation Mapping (CAM). These methods were favored due to their model-agnostic nature and ease of use, enabling interpretable explanations without modifying the original models. Future research should propose new methods and explore the adaptation of other XAI employed in other domains to better suit the unique characteristics of spectroscopic data.

## Introduction

Artificial intelligence (AI) has revolutionized various fields of computer science, and one prominent application is its integration into medical and chemical analysis systems. AI models have been developed in medicine to assist with clinical decision-making, disease diagnosis, and patient data management. One modality often used in the medical domain is spectral techniques, like IR, Raman spectroscopy, and mass spectrometric techniques. Also, for these spectral and spectroscopic measurement techniques, AI has made significant advancements.

The analysis and interpretation of spectral data often involve integrating mathematical, statistical, and chemical measurement methods. In recent years, artificial intelligence (AI) algorithms, including Partial Least Squares regression (PLS) [19], Random Forest (RF) [6], Support Vector Regression (SVR) [10], Linear Regression, Support Vector Machine (SVM) [4], and deep learning, have emerged as powerful tools for handling spectral data. Deep learning algorithms, in particular, can automatically extract relevant features from Raman spectral data, eliminating the need for manual feature engineering. This advancement has significantly improved data modeling, classification, and regression tasks in spectral analysis.

However, the lack of transparency and explainability is a typical limitation of AI models, which is especially important in the medical field and various Raman spectroscopy applications, impeding their acceptance by healthcare professionals and chemical researchers. To tackle this issue, explainable artificial intelligence (XAI) has emerged as a critical research area, aiming to provide understandable and transparent explanations for AI model outputs and operations. XAI bridges the gap between complex AI algorithms and end-users by offering insights into the generation of predictions, enabling greater comprehension and trust in the model’s decisions.

This review provides a pioneering examination of the application of explainable artificial intelligence (XAI) in spectroscopy. It highlights the limited utilization of XAI in this specific domain, as indicated by the relatively small number of conducted studies to date. It further highlights the prevalent trend of adapting or modifying existing methods from other data types, such as images and multivariate data, for spectroscopic analysis. This work aims to enhance our understanding of XAI’s role in spectroscopy and the potential to significantly improve AI models’ reliability, transparency, and acceptance in these fields. The report also addresses the challenges associated with integrating XAI techniques and overcoming the limitations of black box AI models. Moreover, it explores the obstacles involved in data interpretation in machine learning algorithms for spectral analysis.

## Terminology

In this section, we aim to ensure clarity and facilitate comprehension by introducing the terminology used throughout the document. This will provide a solid foundation for understanding the subsequent discussions on XAI techniques specifically applied to spectroscopy-based models.

*Dataset*: tabular representation of data that includes instances $x$ and targets $y$.

*Instance*: a single row or entry in the dataset. Also known as a “sample,” or “observation,” i.e., a Raman spectrum from a substance measurement.

*Input features*: the dataset columns that describe each instance’s attributes, i.e., the Raman intensity at a certain wavenumber. The features provide valuable information about the sample. These characteristics are used for a machine learning model to determine a target value. The vector of a specific feature across all instances.

*Machine learning model*: a program that learns from known data to make predictions or identify patterns. It can be used for classification, regression, clustering, or outlier detection tasks.

*Target*: the information that a machine learning model aims to predict (classification or regression).

*Prediction*: the estimated value for the target variable obtained after evaluating the given features by a machine learning model.

*Black box model*: a type of model characterized by its opaque internal mechanisms, making it difficult to understand by simply examining its parameters, such as neural networks.

*White box*: the opposite of a black box model, a white box model is an interpretable model that can be easily understood and examined, i.e., a linear model.

*Interpretable model*: a model that humans can easily understand, with a clear and straightforward internal decision process. Examples include linear models and decision trees, known for their transparency.

*Linear models*: models that provide a straightforward relationship between inputs and outputs through a linear equation. Each model coefficient represents the contribution of a corresponding feature, making it easy to understand and explain how predictions are made.

*Decision trees*: models that visually represent decision paths and splits based on feature values. Decisions are reached at each node, and the entire decision-making process can be followed by tracing the path from the root to the leaf.

*Interpretability*: this refers to how well a model can be understood by humans. It focuses on the clarity of the model’s internal decision process and how easily a human can comprehend the steps it takes to make decisions. A model is considered interpretable if its operations are simple enough for a human to understand.

*Explainability*: this provides insights into the reasons behind a model’s decisions. It aims to make the model’s decision-making process more transparent and understandable. Explainability tools and techniques aim to break down complex model outputs into understandable components, often through post hoc explanations that clarify the decision-making process.

*Explainable artificial intelligence (XAI)*: a field of study focused on developing methods and models that enable human understanding of the behavior and reasoning behind machine learning systems. XAI aims to create models that provide transparent and comprehensible explanations for their predictions or decisions. By incorporating interpretability into the learning process, these models enhance trust, accountability, and domain knowledge integration, allowing humans to validate, understand, and make informed decisions based on machine learning outputs.

*Global explanations*: provide an understanding of the overall decision-making process of a model, revealing how inputs are transformed into output decisions at the model level. They uncover underlying patterns and mechanisms, providing insights into the model’s behavior across different inputs.

*Local explanations*: focus on explaining specific decisions made by the model. They aim to identify the input features (e.g., pixels in an image, intensity in a spectrum) that positively or negatively contribute to the model’s output for a given instance. Local interpretability techniques estimate scores for each input feature, capturing their impact on the network output. These scores are often visualized as heatmaps, aligning with the dimensions of the input features.

The distinction between explainability and interpretability is a significant topic of interest in the literature, though these terms are sometimes used interchangeably. In this work, we adhere to the definitions of explainability and interpretability as given by Gilpin et al. [14]. Interpretability refers to how well a model can be understood, defined as the ability to clearly describe a system’s internals. Its success depends on the user’s understanding, knowledge, and biases, necessitating simple and meaningful descriptions. Explainability, on the other hand, involves a model’s ability to summarize the reasons for its behavior, gain user trust, and provide insights into its decisions. While explainable models are inherently interpretable, they extend further by justifying their actions and offering relevant responses to questions, such as why a particular decision was made.

Neural networks, particularly those with convolutional layers, can be somewhat interpretable because their mathematical operations can be described and understood. The functions of the convolutional layers are based on well-defined mathematical principles, allowing for a certain level of interpretability. However, a high number of layers, operations, and the optimization process that fine-tunes these networks make it challenging to fully understand how specific decisions are made. Therefore, while neural networks might achieve some degree of interpretability, their overall complexity often requires a focus on explainability. In this context, explainability involves summarizing the network’s behavior and providing insights into its decision-making process, even if the intricate details of the optimization remain unclear.

The terminology and definitions surrounding explainability and interpretability may evolve and change with time. However, this manuscript considers explainability more important and comprehensive than interpretability. Interpretability is a crucial first step but only partially addresses understanding opaque models. Explainability aims to provide a high level of detail (completeness) and communicate this detail effectively (interpretability). The challenge is to create explanations that balance these aspects, avoiding overly simplified descriptions that could mislead users while ensuring explanations are detailed enough to be trustworthy and informative.

## Literature selection

The existing literature includes review studies that have explored the use of XAI in natural and medical image analysis [2, 3, 15, 16, 26–30]. However, there is a clear need for a comprehensive systematic review that explicitly investigates the application of XAI in spectroscopy. In line with this need, our study aims to conduct a systematic review encompassing various measurement techniques and dataset types. By doing so, we will provide a comprehensive overview of the current landscape in this field, addressing the potential benefits and challenges associated with employing XAI in spectroscopy.

**Fig. 1:**
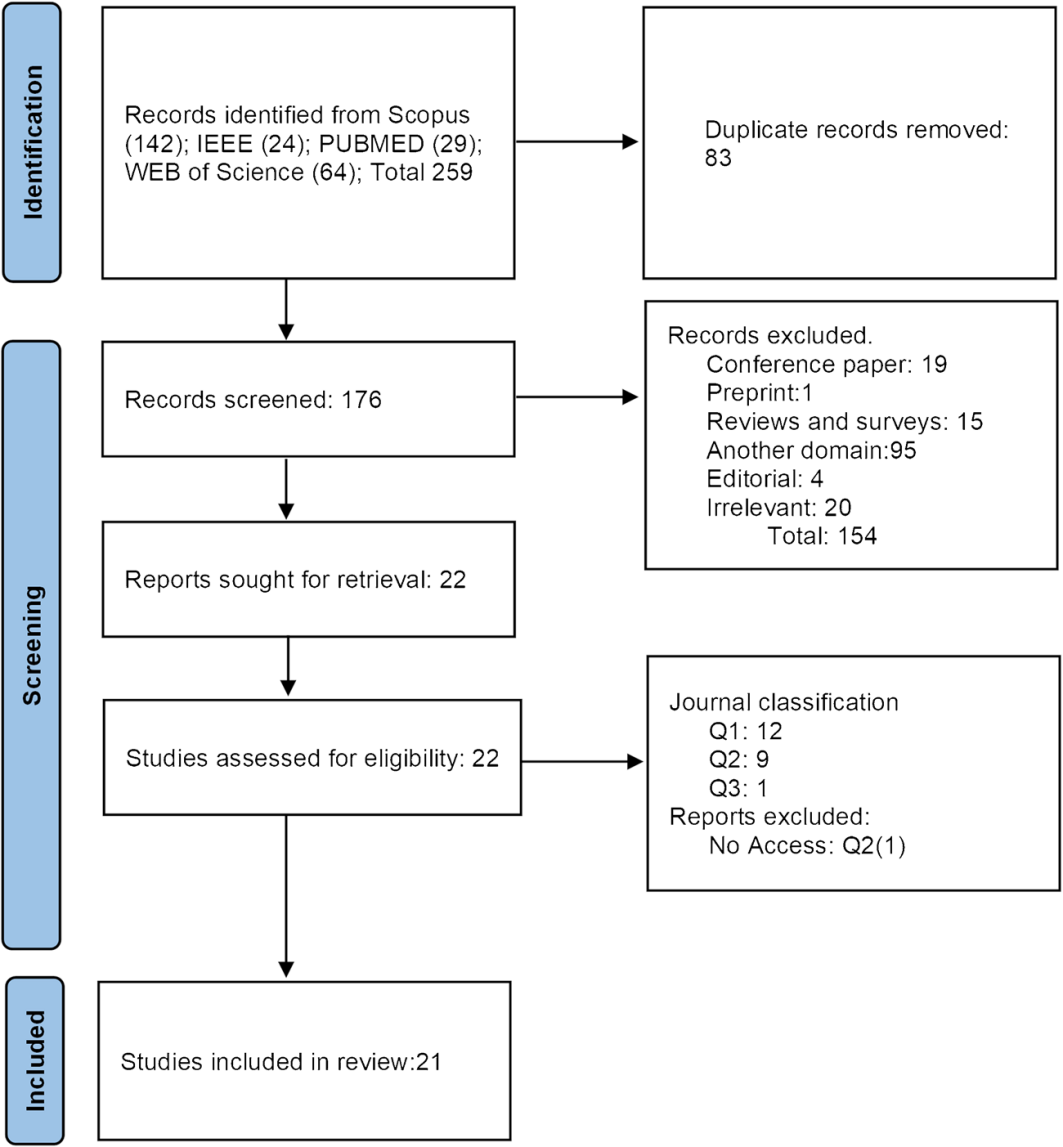
PRISMA systematic filtration of journal articles

Our review study employed the PRISMA guideline 2020 [30] to systematically filter our search results. Initially, we obtained 259 search results from journal databases such as Scopus, Institute of Electrical and Electronics Engineers (IEEE), PubMed, and Web of Science. Utilizing the Boolean search strings presented in Table 1, we narrowed our search to include publications until June 2023. To ensure the integrity of our study, we removed 83 duplicate publications across the journal databases, as illustrated in Fig. 1. Next, we screened the titles and abstracts of the remaining publications, excluding conference papers, pilot studies, non-journal articles, other review and survey studies not focused on spectrometry, editorials, and studies not relevant to XAI or spectrometry.

**Table 1:** Boolean search strings and number of studies retrieved from respective journal databases

Database	Boolean search strings
Scopus search (141)	TITLE-ABS-KEY ( ( explainability OR explainable)) AND ( TITLE-ABS-KEY ( ( machine AND learning) OR ( deep AND learning))) AND TITLE-ABS-KEY ( ( raman OR spectroscopy OR spectra OR spectral OR spectrum OR chemometrics OR cars OR sers)) AND ( LIMIT-TO ( DOCTYPE, "ar"))
IEEE (24)	("Abstract":"explainable" OR "Abstract":"explainability") AND ("All Metadata":"machine learning" OR "All Metadata":"Abstract":"deep learning") AND ("All Metadata":raman OR "All Metadata": spectroscopy OR "All Metadata": spectra OR "All Metadata": spectral OR "All Metadata": spectrum OR "All Metadata": chemo-metrics OR "All Metadata": cars OR "All Metadata": sers)
PubMed (29)	explainability[Title/Abstract] OR explainable[Title/Abstract]) AND (machine learning[Title/Abstract] OR deep learning [Title/Abstract]) AND (raman[Title/Abstract] OR spectroscopy [Title/Abstract] OR spectral [Title/Abstract] OR spectrum[Title/Abstract] chemometrics [Title/Abstract]
Web of Science (64)	(AB = (explainable) OR AB = (explainability)) AND (AB = (machine learning) OR AB = (deep learning)) AND (AB = (raman) OR AB = (spectroscopy) OR AB = (spectral) OR AB = (spectrum) OR AB = (chemometrics))

This screening process resulted in 22 scientific studies being considered for inclusion in our review. Furthermore, considering the limited number of results, we decided not to exclude articles from journals classified as Q2 and Q3 based on the journal quartiles obtained from the scientific journal ranking available at https://www.scimagojr.com/journalrank.php. Furthermore, it is essential to note that one article was inaccessible [39]. As a result, we retained 21 scientific articles, as one article was not accessible to us. These twenty-one articles included those from Q1 journals renowned for their high credibility, as well as those from Q2 and Q3 journals.

## Result and discussion

Given the recent introduction of explainable artificial intelligence (XAI), it is not surprising to observe a limited number of publications in the field of spectroscopy. Furthermore, the fact that the earliest publication dates to 2020 suggests that XAI has only recently gained recognition in high-impact journals, indicating its growing popularity.

It is anticipated that the number of publications in this area will increase in the coming years as interest in XAI continues to grow. Table 2 provides an overview of the various XAI methods employed in these studies, with some papers utilizing multiple methods. Among the most widely used XAI techniques are SHapley Additive exPlanations (SHAP) [25], masking methods inspired by Local Interpretable Model-agnostic Explanations (LIME) [32], and Class Activation Mapping (CAM) [34, 50] and Gradient-based methods.

**Table 2:** Distribution of XAI methods and visualization outputs in reviewed studies, some employing multiple methods. Describing visualization types and their interpretive value for model predictions and behaviors

Method	Papers	Working principle/category	Visualization plot/output	Description
SHAP	9	Perturbation-based/gradient-based	Force plots	Visualize the contribution of each feature to the prediction for individual instances
			SHAP summary plots	Global view of feature importance and the impact of features on model output across the entire dataset
			SHAP waterfall plot	Sequentially illustrates each feature’s contribution to a model’s final prediction, starting from a base value
Masking methods/LIME	3	Perturbation-based	LIME explanations	Local interpretable explanations showing feature importance in the form of weighted features
			Feature importance bars	Bars or pie charts showing the importance of each feature for the specific prediction
**CAM/**GRAD-CAM	4	Activation-based/gradient-based	Heatmaps	Highlighting key image areas for predictions by overlaying them, emphasizing “activated” regions
			Activation maps	Visual representations of CNN layers, showing how certain features activate on the given input
Gradient-based	4	Gradient-based	Saliency/attribution maps	Emphasize key features; intense colors indicate greater impact on the model’s output
Global surrogate	1	Surrogate-based	Feature importance	Global feature importance representing the overall importance of features in the model
			Partial dependence	Illustrate feature-outcome connection, considering the average of other features’ distributions
Activation map embedding	3	Activation-based	t-SNE or UMAP visualizations	High-dimensional activation is reduced to lower-dimensional space for visualizing input impact
			Feature map	Visualize activation value distributions in specific layers or neurons
Total papers	24			

**Fig. 2:**
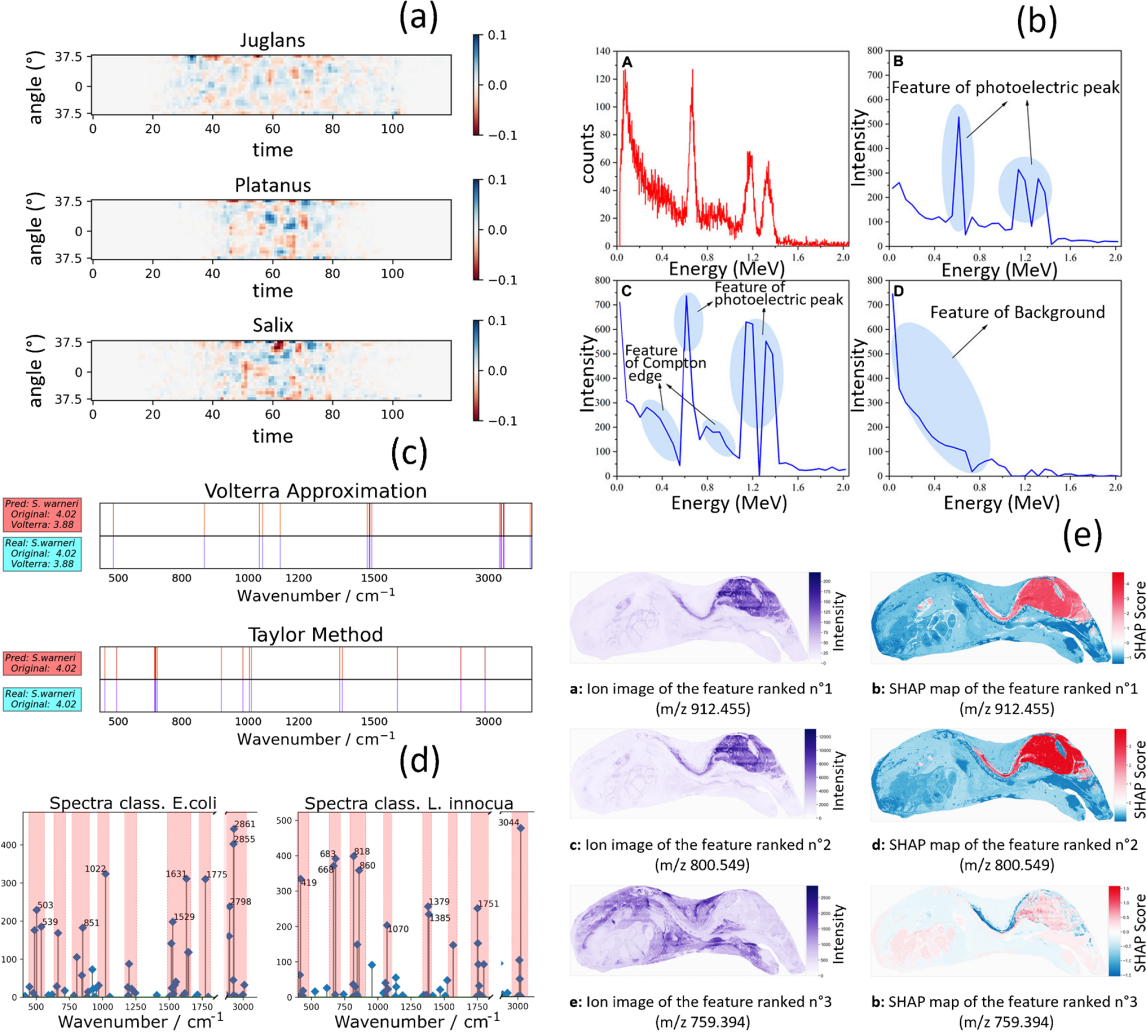
**a** Gradient-based method, attribution maps show color-coded contributions and how different scattering data points contribute to predicting pollen classes, with blue indicating a positive effect and red a negative one. Image adapted from “Explainable AI for unveiling deep learning pollen classification model based on fusion of scattered light patterns and fluorescence spectroscopy” by Brdar et al., CC BY, 2023. **b** Activation maps, feature maps (B, C, D) highlighting specific spectral features. (A) original gamma-ray spectrum of mixed nuclide spectra from cobalt-60 and cesium-137. Image adapted from “Multiple radionuclide identification using deep learning with channel attention module and visual explanation” by Wang et al. CC BY, 2022. **c** Global surrogate, saliency maps reveal the specific input regions the model prioritizes when making predictions. (Top) Volterra approximation. (Bottom) Taylor-based method. Image adapted from “Agnostic eXplainable Artificial Intelligence (XAI) Method Based on Volterra Series” by Contreras and Bocklitz. CC BY-NC-ND, 2023. **d** Global surrogate, feature importance plots highlight the areas most influence the model output. Image adapted from “Agnostic eXplainable Artificial Intelligence (XAI) Method Based on Volterra Series” by Contreras and Bocklitz. CC BY-NC-ND, 2023. **e** SHAP, SHAP maps on the right offer insights into feature relevance for brain recognition, while ion images on the left show feature distribution and intensity. Image adapted from “Automated biomarker candidate discovery in imaging mass spectrometry data through spatially localized Shapley additive explanations” by Tideman et al. CC BY. 2021

**Fig. 3:**
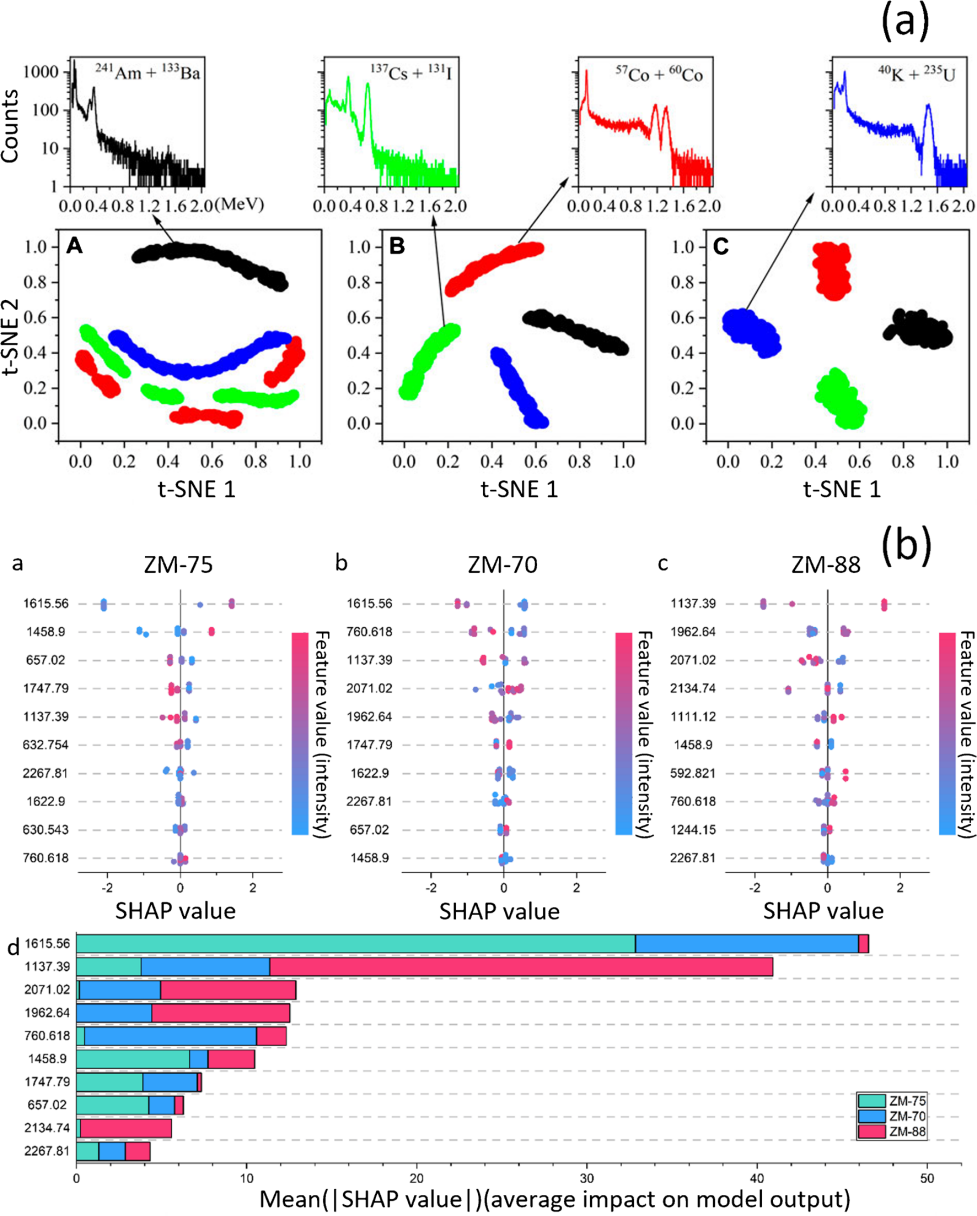
**a** Activation map embedding, feature visualization using t-SNE: (A) visualization of channel max-pool outputs, (B) global average pooling 1d, (C) dense layer. Image adapted from “Multiple radionuclide identification using deep learning with channel attention module and visual explanation” by Wang et al. CC BY. 2022. **b** SHAP, (a–c) SHAP value distributions for Raman wavenumbers in cottonseed cultivars ZH-75, ZH-70, and ZH-80. (d) Average impact of the top 10 most influential Raman wavenumbers. Image adapted from “Insights into Cottonseed Cultivar Identification Using Raman Spectroscopy and Explainable Machine Learning” by Chi et al. CC BY. 2023

The techniques listed in Table 2 can be classified into distinct categories: activation-based, perturbation-based, gradient-based, surrogate-based, and various combinations thereof. Furthermore, Table 2 includes an outline of the types of visualization plots or outputs each method typically generates, along with brief descriptions to illustrate how these visualizations aid in interpreting and understanding model predictions and behaviors. Figures 2 and 3 offer a graphical illustration of the outcomes generated by some XAI methods. To ensure that research objectives and goals are aligned, it is crucial to select an appropriate technique. Below, the most popular XAI techniques utilized for spectroscopy-based models are introduced with a concise explanation that provides an overview of their underlying principles, objectives, advantages, and disadvantages. We also present existing implementations providing valuable insights into XAI’s implications and potential advancements in spectroscopy.

### Activation maps

Activation maps, or feature maps or heatmaps, are direct and intuitive techniques used to visualize the spatial activation patterns of neurons in a specific layer of a network in response to an input. By analyzing these maps, it is possible to understand which regions or features in the input are being detected and activated by the network. Without any additional techniques, it can still provide some insights into the decision-making process of a neural network. Activation maps have limitations, such as the absence of quantitative information, making it difficult to measure the precise impact of features on the model’s decisions. Deeper layers can also lead to more abstract representations, reducing the comprehensive analysis of complex models. Nonetheless, activation maps remain valuable for identifying areas of improvement, debugging models, and uncovering potential biases.

Tsimpouris et al. [43] utilize near-infrared diffuse reflectance spectroscopy at 350–2500 nm and a CNN to predict eight properties in mineral samples. Activation maps of the embedding feature are utilized to enhance the interpretability of transformed feature spaces and compressed spectra, providing valuable insights into the trained model. The highest and lowest activation patterns were identified and visually compared, considering their spectral signatures and related properties.

Wang et al. [47] investigate the application of attention mechanisms and channel attention modules in a CNN for multiple radionuclide identification. The research showcases activation maps extracted from different convolution kernels in the final convolutional layer, highlighting essential aspects such as photoelectric peaks, the Compton edge, and the background in gamma spectroscopy. Additionally, t-distributed stochastic neighbor embedding (t-SNE) [17] was used for visualizing the feature extraction outcome of the convolutional blocks. t-SNE effectively shows complex relationships in high-dimensional data with multiple manifolds.

### Perturbation-based

Perturbation-based XAI methods involve perturbing or altering input data to observe the resulting changes in the output of a model, aiming to understand the model’s decision-making process through the analysis of input perturbations. Analyzing the effects of input perturbations provides valuable information into the internal processes of complex models, relieving feature importance and model bias. Perturbation-based methods can provide local explanations specific to individual instances, which aids in identifying potential biases or robustness issues.

However, perturbation-based methods may suffer from certain drawbacks, like potential prediction effects, lack of physical sense with specific data types, and computational expense in generating numerous perturbed samples for complex models. These methods focus on local approximations, making them insufficient for capturing the model’s overall behavior for certain applications. In spectroscopy, the distinctive data nature permits combining local solutions for a global one. The consistent alignment of wave numbers facilitates direct comparisons and insight integration, enabling a comprehensive analysis of diverse samples and materials.

Two commonly used methods in this category are Local Interpretable Model-agnostic Explanations (LIME) [32] and SHapley Additive ExPlanations (SHAP) [25]. LIME generates perturbed samples around a specific instance and trains an interpretable model on these samples to approximate the model’s behavior locally and provides explanations specific to individual instances. On the other hand, SHAP assigns values to each feature in a prediction using game theory concepts. By perturbing feature combinations and observing the model’s output, it calculates the contribution of each feature to the prediction. SHAP is the most widely used method in spectrometry for XAI. Therefore, we will give it a separate subsection to provide a more in-depth explanation.

Wang et al. [44] predict solution conductivity by utilizing a Neural Network (NN) to characterize it using optical emission spectroscopy (OES) of plasma in an aqueous solution. LIME was employed to interpret the NN by dividing the spectra into six segments, representing the six emission lines, and selectively retaining or masking these segments to perturb the spectra. LIME evaluates the contributions of each emission line by fitting a linear model to the perturbed features, thus facilitating an understanding of their significance and impact.

Li et al. [24] employ a SENet-LSTM [20] model and Raman spectroscopy to analyze gastric adenocarcinoma (GAC) samples and classify between cancerous gastric mucosa and normal gastric mucosa. A masking method inspired by LIME is used to identify relevant regions in Raman spectra for the model. It preserves the *i*th dimension while masking out others for predictions. Accuracy and loss values are recorded for each dimension. Iteratively, dimensions with high accuracy and low loss are selected, forming a sequence of important regions. This process continues until five consecutive points no longer reduce prediction accuracy, yielding the final sequence. Later, important regions are assigned weights using step-by-step propagation. From these identified regions, points are sequentially selected and assigned weights with decay around critical points. After crossing the entire sequence, each important point is assigned a final weight.

### SHapley Additive exPlanations

SHapley Additive exPlanations (SHAP) is the most widely used perturbation-based XAI approach. It is a model-agnostic explainer that calculates the contribution of each feature to the prediction of any machine learning model based on the game theory concepts [25]. It introduces two notable advancements compared to Shapley values [35]. Firstly, the authors introduced KernelSHAP, which offers an alternative method to estimate Shapley values by using kernels and drawing inspiration from local surrogate models. Secondly, SHAP encompasses various global interpretation methods that involve aggregating Shapley values, expanding its interpretability beyond individual predictions. The Shapley value (*ϕ*_i_) represents the fair average contribution of a feature value across all possible coalitions, which are different subsets of features formed by considering different combinations. It represents the importance of a feature by measuring its average contribution across these coalitions, providing valuable insight into the overall model gain. It is important to note that the Shapley value does not indicate the prediction difference resulting from removing a specific feature but rather the average contribution of a feature value in various coalitions. However, calculating Shapley values can be computationally intensive, especially for large datasets or complex models, which may hinder its scalability in specific scenarios. Moreover, while SHAP provides valuable insights into feature importance, it does not directly indicate the effect of removing a particular feature on the model’s prediction, limiting its capacity for causal analysis.

Chi et al. [8] developed a Raman spectroscopy analysis framework with three machine learning algorithms (Support Vector Machine, eXtreme Gradient Boosting [7], and Neural Network) to identify cottonseed cultivars. Moreover, SHAP was employed to enhance interpretability, focusing on crucial Raman peaks and understanding the contributions of features in the identification process.

Kalopesa et al. [22] aim to predict sugar content in wine grapes employing VNIR–SWIR Point Spectroscopy and machine learning, Random Forest regression (RF) [6], Partial Least Squares regression (PLS) [19], Support Vector Regression (SVR) [10], and CNN. Various analyses were performed to identify the key wavelengths necessary for estimating the sugar content in wine grapes. The Variable Importance in Projection (VIP) [12] scores were plotted for PLS, while the Gini score [29] was visualized for RF. The Shapley Additive Global importance (SAGE) values were plotted for SVR and CNN. These last exhibited sparse patterns with sharp peaks, indicating the presence of crucial features.

Nakanishi et al. [27] utilize an extremely randomized tree regressor [13] to predict cell density in autotrophic/heterotrophic microorganism mixtures. The model employs absorbance spectrum data obtained from a mixture of *Saccharomyces cerevisiae* and *Chlamydomonas reinhardtii*. Additionally, SHAP was employed to assess feature importance.

Shibu et al. [36] proposed xAI-fNIRS, an XAI model developed for predicting brain states using functional near-infrared spectroscopy (fNIRS) signals, able to distinguish between brain states. xAI-fNIRS comprises a classification module and an explanation module. The classification module includes a CNN and a long short-term memory (LSTM) [18] module. The explanation module utilizes SHAP to identify the most influential channels, their locations, and the corresponding oxy- or deoxy-hemoglobin levels.

Singh et al. [38] focus on estimating the nitrogen content in wheat by utilizing six different machine learning techniques. The spectral reflectance, obtained through hyperspectral data, is utilized to predict the nitrogen status of plants. Moreover, the study incorporated SHAP values to provide insights into the factors influencing the nitrogen estimation process.

Tideman et al. [42] present a method for identifying biomarker candidates in imaging mass spectrometry (IMS) data based on their mass-to-charge ratios. An XGBoost model is utilized for classification, and SHAP values are employed to estimate the relevance of specific biological classes quantitatively. The method identifies potential biomarkers and provides insights into their spatial distribution and relevance.

Wen et al. [48] propose an explainable machine learning approach employing Random Forest regression (RFR) to predict the coal ash content of samples using X-ray fluorescence (XRF) composition data. SHAP is utilized to interpret the model and determine the importance of different elements in predicting ash content. The SHAP interpretation reveals that the nine most important elements for ash content prediction are Al, S, Si, Fe, Ca, Ti, K, Sr, and Zr.

### Gradient-based methods

Gradient-based method leverages the gradients of the network output (logits or SoftMax probabilities) with respect to the input features to estimate the attribution map [37]. This map provides insights into the relevance of each input feature for a particular output class, highlighting their respective importance in the decision-making process. To enhance the visualization of the attribution map, one can apply either absolute values or a normalization strategy. This can involve ignoring negative values or values below a specific threshold. By employing such a normalization strategy, the visualization of the attribution map can be improved, emphasizing the most relevant features while minimizing the influence of irrelevant or insignificant values.

Gradient-based might not always provide a complete understanding of complex model behavior, especially in cases where features interact nonlinearly or when dealing with deep and intricate architectures. Furthermore, these methods can be sensitive to input variations, potentially leading to noisy attributions, which may affect the overall interpretability.

Brdar et al. [5] propose an explainable framework for pollen classification using DL. Three data sources, namely scattering, spectrum, and lifetime, are utilized to capture the optical fingerprints of pollen particles. The assessment of morphological properties involves the analysis of light scattering, while the encoding of chemical properties is achieved by examining the fluorescence spectrum and lifetime. The critical features contributing are examined by considering domain knowledge and comparing them with reference data. Instance-level explanations are generated using the integrated gradient method [41]. For the model-level explanations, the top-ranked features influencing the decision process are highlighted.

Joung et al. [21] focus on developing a AI model to predict organic chromophores’ optical and photophysical properties. Integrated gradients are employed to obtain attributions, which reveal the contributions of functional groups to various optical properties, including absorption wavelength, bandwidth, extinction coefficients, emission wavelength, bandwidth, photoluminescence quantum yield, and lifetime.

Panos et al. [31] utilize XAI to identify preflare spectral features for predicting solar flares using a CNN. The Expected Gradients (EG) [11] and gradient-weighted Class Activation Mapping (Grad-CAM) [34] techniques are employed to analyze Mg II spectra from flaring and non-flaring active regions. Grad-CAM and EG establish the equivalence between integrated attributions and prediction scores, providing insights at the wavelength scale. For global explanations, regions around the Mg II h&k line cores and triplet emission are highlighted, revealing CNN’s preference for features such as red and blue wing enhancements and broad spectral cores. Additionally, the study finds that downflows receive more attention than upflows in the dataset.

Zhang et al. [49] employ a multi-task U-Net framework to investigate hyperspectral imaging (HSI) for liver tumor delineation on surgical specimens. It combines gradient backpropagation with spectral channel selection to generate saliency maps in hyperspectral images. A saliency-weighted channel selection technique is introduced, identifying a subset of five spectral channels from the original 224 channels. The explanations of the contributions emphasize the significance of individual pixels and channels.

### Class activation mapping

Class Activation Mapping (CAM) [50] is a visualization technique utilized to identify the significant regions within an image that contribute to the predictions made by a CNN. By analyzing the activations of the final convolutional layer, CAM associates these activations with the predicted probabilities of different classes, thereby identifying spatial locations relevant to each class. CAM produces a single heatmap by combining weighted feature maps from the final convolutional layer. This approach facilitates the interpretation and understanding of which input regions are most influential in CNN’s prediction for a particular class. Another notable technique is gradient-weighted Class Activation Mapping (Grad-CAM) [34], which offers visual explanations for CNN decisions by examining activation patterns in the final convolutional layers. By generating a coarse localization map, Grad-CAM highlights the relevant regions of the input. It assigns a relevance score to each neuron based on the specific interest decision. Unlike traditional backpropagation methods that propagate information to the input, Grad-CAM backpropagates this information only to the last convolutional layer. These techniques prove especially valuable for tasks where spatial localization is critical. However, they may encounter limitations when applied to complex models. Additionally, the quality of explanations heavily depends on the model’s architecture and input data.

Kim et al. [23] introduce a CNN that prioritizes interpretability and explanation over classification performance. This model identifies bacterial species based on mass spectral fingerprints without preprocessing steps, including a global average pooling layer that replaces the fully connected layers. The proposed structure generates class activation maps, illustrating the weighted activation maps for each spectrum, and exhibits scale-invariant properties suitable for diverse bacterial spectra.

Wang et al. [45] explore the application of a CNN in the real-time classification of volatile organic compounds (VOCs) through the analysis of optical emission spectroscopy of plasma. The CNN model is interpreted using Grad-CAM, generating feature maps from the testing dataset, and presenting them in the average form. Real-time and online monitoring of VOCs is achieved, accompanied by instant warning messages upon VOC detection. Grad-CAM leverages the gradient of the CNN output for the feature maps of the last convolutional layer, enabling the visualization of the contribution of each spectral feature.

Wang et al. [46] propose an explainable approach for radionuclide identification using gamma-ray spectra, CNN and CAM. CAM analyzes the model and the influence of the spectra with different characteristics, generating saliency maps that highlight distinctive regions. These maps aid in interpreting and identifying specific radionuclides.

### Global surrogate

Global surrogate models are employed to approximate the predictions of complex machine learning (ML) models with more straightforward and interpretable models. The purpose of the surrogate model is to mimic the overall behavior of the black box model without requiring any specific knowledge of its internal workings, making it model-agnostic. Predictions of the black box model are obtained for a given training dataset to train the surrogate. Then, an interpretable model such as Linear Regression, Decision Tree, or K-nearest neighbor is trained using the black box predictions as targets. The choice of the interpretable model allows for insights into the behavior of the original model through the coefficients or other interpretable properties. The surrogate model’s performance in capturing the black box model’s behavior is evaluated by measuring the error between their respective predictions. It is crucial to highlight that the black box model’s performance does not influence the training of the interpretable surrogate model in accurately predicting the actual outcome.

However, global surrogate models have limitations; they may only capture some complexities, especially in cases with highly non-linear or multi-layered black box models. The quality of the surrogate model heavily depends on the quality and representativeness of the training data used to obtain predictions from the black box model. Insufficient or biased training data could lead to inaccurate or misleading surrogate models.

Akulich et al. [1] use several ML techniques, including Linear Regression, SVM, and NN regressions to predict the ignition properties, specifically the Cetane number, of fuel for Unmanned Aircraft Systems (UAS) propulsion systems. This work also employs some interpretable techniques, such as LIME, SHAP, and a global surrogate (GS) [40]. This GS considers feature interactions and perturbs all feature subsets to compute the mean situational importance of each feature’s value. Additionally, the method enhances efficiency by incorporating quasi-random and adaptive sampling techniques in the sampling algorithm. These XAI methods are employed to gain information about the decision-making process, to identify crucial regions within spectroscopy data, and to optimize data collection by miniaturizing the spectrometer device. This optimization reduces device size, lowers power consumption, and saves costs.

Following the systematic search process guided by the PRISMA 2020 guidelines, we identified a single paper implementing this concept. However, during our research, we also encountered a pertinent conference paper that will be referenced, although it will not be included in the analysis presented in Table 1.

Contreras and Bocklitz [9] propose a surrogate model called Volterra-XAI, based on the Volterra series, which approximates a black box model. The proposed method is evaluated in the context of Raman spectrometry for bacteria classification using two CNN architectures. Volterra-XAI incorporates the second-order Volterra layers to extract relevant information for generating saliency maps that explain the contributions of input elements to predictions. The reliability of the visualizations was assessed by comparing the Volterra approximation with the original model.

## Discussion and conclusions

Recent discussions in the literature, such as those by Rudin [33], argue that rather than explaining black box models, the priority should be developing interpretable models from the beginning. This perspective emphasizes the value of creating models that are inherently understandable and transparent. The drawback of this argument is that new, more complex CNN systems often outperform easier and interpretable models. However, Explainable AI can still be integrated to develop better models. For instance, XAI-GAN (Explainable AI Generative Adversarial Networks) [26] is designed to perform well in generating data and provide insights into how and why it generates specific outputs, making them both interpretable and explainable.

Integrating XAI techniques to improve model development and understanding could benefit spectroscopy-based models. This integration would allow researchers to gain deeper insights into spectral data and enhance result reliability. Accordingly, in this study, a total of 22 studies were reviewed, the source code for the most used XAI techniques can be found in the following:

- SHAP https://github.com/slundberg/shap.
- Grad-CAM https://github.com/ramprs/grad-cam/.
- LIME https://github.com/marcotcr/lime.

Furthermore, it is worth noting that most studies utilize SHAP and masking methods. These approaches are preferred due to their model-agnostic nature, which means they do not require any modifications to the original model. Moreover, their ease of use adds to their popularity in XAI literature.

While most XAI methods used for spectral data primarily focus on identifying specific intensity peaks, a few notable exceptions [24, 44] diverge from the conventional approach used in multivariate data analysis. These exceptional works emphasize the importance of considering spectral bands and recognize that prioritizing the influence of these bands is crucial to achieving consistent and reliable results, as opposed to solely relying on individual intensity values. We believe that the influence of spectral bands should be given greater importance, which helps ensure consistent results that may arise from considering individual intensity values.

This study has focused on XAI methods applied to spectroscopy; it is essential to acknowledge that other XAI methods are commonly employed in image analysis and multidimensional data that have yet to be explored within the spectroscopy domain, which are worth investigating further. It is worth noting that all the methods examined in this study originate from image and multivariate analysis techniques. However, it is crucial to recognize that spectra possess unique characteristics that distinguish them from multidimensional data or signal processing. Consequently, it is essential to exercise caution when directly applying specific methods without considering these distinctive characteristics, as they can significantly impact the effectiveness and reliability of the results obtained. Future research should explore and adapt other XAI methods to better suit the intricacies of spectroscopy data and uncover novel insights in this field.

## Data Availability

Not applicable.
